# Successes and Challenges of Fostering Age-Friendly Health Systems Through a Long-Term Services and Supports ECHO

**DOI:** 10.1093/geroni/igaa057.1722

**Published:** 2020-12-16

**Authors:** Cherie Brunker, Jorie Butler, Jacqueline Telonidis, Yin-Shun Chiu

**Affiliations:** 1 University of Utah, Salt Lake City, Utah, United States; 2 University of Utah - College of Nursing, Salt Lake City, Utah, United States

## Abstract

Launched in June 2016, the Utah Geriatric Education Consortium (UGEC) inter-professional Learning Community was developed by faculty in collaboration with primary care and community partners with a focus on Long Term Care. In 2019, UGEC expanded to include Assisted Living, Home Care and Hospice -5 primary care and 6 community long-term services and supports (LTSS) partners in rural and urban Utah with a primary objective of developing and training existing LTSS healthcare providers and direct care workers on the elements of age-friendly health systems (“4Ms”: mentation, medication, mobility, and what matters) to enhance care and improve outcomes for older adults across the continuum of LTSS. Attendance in the monthly distance learning sessions averages 27.5. Sessions are rated highly and engage nurses, behavioral health workers, administrators, physicians, nursing assistants, occupational and physical therapists. The process, challenges, successes of transitioning to a LTSS ECHO (Extension for Community HealthCare Outcomes) will be discussed.

